# Vehicular Localization Enhancement via Consensus

**DOI:** 10.3390/s20226506

**Published:** 2020-11-14

**Authors:** Hong Ki Kim, Minji Kim, Sang Hyun Lee

**Affiliations:** School of Electrical Engineering, Korea University, Seoul 02841, Korea; istackcheese@korea.ac.kr (H.K.K.); kimminji1013@korea.ac.kr (M.K.)

**Keywords:** autonomous vehicular networks, cooperative localization, vehicle-to-everything (V2X), consensus algorithm

## Abstract

This paper presents a strategy to cooperatively enhance the vehicular localization in vehicle-to-everything (V2X) networks by exchanges and updates of local data in a consensus-based manner. Where each vehicle in the network can obtain its location estimate despite its possible inaccuracy, the proposed strategy takes advantage of the abundance of the local estimates to improve the overall accuracy. During the execution of the strategy, vehicles exchange each other’s inter-vehicular relationship pertaining to measured distances and angles in order to update their own estimates. The iteration of the update rules leads to averaging out the measurement errors within the network, resulting in all vehicles’ localization error to retain similar magnitudes and orientations with respect to the ground truth locations. Furthermore, the estimate error of the anchor—the vehicle with the most reliable localization performance—is temporarily aggravated through the iteration. Such circumstances are exploited to simultaneously counteract the estimate errors and effectively improve the localization performance. Simulated experiments are conducted in order to observe the nature and its effects of the operations. The outcomes of the experiments and analysis of the protocol suggest that the presented technique successfully enhances the localization performances, while making additional insights regarding performance according to environmental changes and different implementation techniques.

## 1. Introduction

The realization of autonomous vehicular networks has been rendered achievable and imminent due to the recent advancements in various communication application areas, including fifth generation (5G) mobile communication and millimeter-wave (mmWave) technologies [1]. To ensure reliable operation of autonomous vehicles, precise and quick localization techniques are in demand. However, the inevitable occurrences of measurement and communication noises often digresses location estimates from the ground truth locations. Furthermore, the usage of Global Positioning System (GPS) has technical limitations since its signal power budget often fails to become sufficient enough to penetrate obstacles; unfavorable environments such as underground tunnels or indoors can lead to more inaccuracy [2]. Detection techniques where the vehicles cooperatively utilize each other’s estimates to enhance the preciseness of the localization are therefore solicited [3].

Cooperative localization can be realized in centralized or distributed manners [4]. The centralized localization is implemented by employing a single computationally superior unit to estimate the locations of all vehicles within the network. Such an approach can be very efficient in various ways since most vehicles’ loads on the computation and communication are significantly lifted. However, the centralized approach may not be robust to communication setbacks since the failure in the centralized management ends up with the eventual collapse of the entire system. In applications such as V2X networks where a brief collision may result in major accidents, a robust and flexible approach is desirable. Furthermore, a centralized manner of the localization has limitations in scalability since the computational loads are devoted solely to a single unit. In such cases, the amount of computation scales directly to the number of the vehicles in the network. Considering the imminent implementation of large-scale vehicular networks, the limit in scalability renders the reliance to a single unit unsuitable. On the other hand, a distributed localization conducts the estimation independently and locally among neighboring vehicles. Since such approach does not require a central agent, it is less reliant to a single unit and easier to increase the network scale. A distributed implementation is therefore preferred in many circumstances, in particular, of mobile network configurations, such as V2X networks.

To that end, distributed localization techniques with data-driven parameter estimation, such as maximum a posterior estimation [5,6,7,8], Monte Carlo (MC) methods [9], maximum likelihood (ML) estimation [10], particle filters (PFs) [11], extended Kalman filters (EKFs) [12,13], and sum-product algorithm over a wireless network (SPAWN) [14], have been developed. Factor graph models [15] and belief propagation (BP) algorithms are utilized in References [5,6,7,8] to reduce communication and computation demands for synchronized cooperative localization. In Reference [9], a modified MC localization is utilized to estimate locations of robots with the robustness in indoor environments. ML estimation combined with numerical optimization techniques is used for localization without external localization units in Reference [10]. In Reference [11], the localization is achieved by combining results obtained from the PF technique and the GPS-based localization. In Reference [16], multi-node cooperative localization is developed based on dead reckoning and PF. With the data fusion of redundant data from multiple sensors, References [12,13,17] implement EKFs and PFs to cooperatively localize a large group of mobile robots and vehicles. In Reference [18], mmWave is utilized for the cooperative positioning while also taking advantage of radio-based multipath signals to build a map of available signal paths. A localization approach based on the SPAWN [14] and BP algorithms repetitively calculates the posterior distributions of messages to achieve localization with high accuracy. A new distributed optimization technique based on alternating direction method of multipliers (ADMM) [19] is also developed to lift a large amount of the computational cost in cooperative localization by updating the solution by using only average values of probability distributions. However, considering the limited amount of computational resources available in a vehicle and the demand for the real-time update on the location, an enhanced protocol with improved localization accuracy is desired.

To overcome these challenges, this work aims at a simple strategy of improving the accuracy of vehicular localization. The strategy is composed of two main steps—updates based on consensus and compensation. In the first half of the update phase, individual vehicles repeatedly, but not necessarily synchronously, exchange and update their location estimates in a consensus-based manner. The consensus-based protocol averages the local estimates by exchanging location data and measuring relative measurement with respect to each other. The iterative updates thus cause all neighboring vehicles to possess similar magnitudes and orientations of the localization error. Since the repetitive updates average out the local estimate errors, it is noticeable that the localization of the best-performing vehicle, called anchor, is bound to degrade. Such an observation is taken into advantage in the second half, the compensation phase, of the proposed strategy. In the compensation step, the homogenized measurement errors are significantly removed by counteracting the degradation of the location estimate of the best-performing vehicle among the neighboring vehicles. The entailed computations are maintained as simple as basic arithmetic operations and evaluation of $L_{2}$ norm. In regard to the communication loads, the iterative data exchanges among the vehicles and the broadcast for the compensation step are involved. These loads can be minimized by communicating the information associated with a sufficient statistic that leads to the resulting estimate achieving Cramér-Rao lower bound (CRLB) [20].

The major contributions of the paper are summarized as:

- An uncomplicated strategy to enhance localization performance in vehicular networks is constructed. The strategy involves the exchange of local data and the exploitation of a superior anchor vehicle within the vehicular network. Using local estimates of different qualities, the proposed strategy enhances the overall accuracy of all vehicles’ localization to that of the anchor. The execution of the protocol does not involve a central manager, thus greatly improving the adaptability of the protocol to networks of different scales.
- Mathematical formulations for the analyses of the different stages of the proposed strategy are made. Despite the computational simplicity, the analytically found optimal estimator efficiency is ultimately achieved in terms of CRLB.
- Visual representations of the experiments based on 3GPP TR 37.885 urban scenario specifications are provided and discussed. The effects of the execution of the proposed strategy are observed under diverse scenarios. Comparisons to centralized localization are also made in order to show the effectiveness of the distributed protocol for the intended environments.

The remaining parts of the paper are organized as follows—Section 2 describes the system model. Section 3 develops a decentralized strategy of improving the localization performance along with performance analysis details. In Section 4, the utilization of the proposed strategy under a representative configuration is observed with discussion. The efficiency of the proposed strategy under various circumstances and implementation practices are addressed in Section 5. Section 6 concludes the paper.

## 2. System Model

In a local vehicular ad-hoc network, $N_{v}$ vehicles communicate with each other via utilizing mmWave technology features. By the utilization of a set of mmWave transceivers that work in all angles, the communications is not affected by the respective orientations of vehicles. Furthermore, as the localization takes place with respect to an instance of time, the vehicles’ mobility causes no direct impact to the connection between the vehicles. However, some pairs of vehicles may be beyond each other’s communication range if the network size is relatively large. The vehicles estimate the locations of themselves by means such as global navigation satellite system (GNSS). Among them, a vehicle that possesses relatively accurate means for self-localization is referred to as an anchor, while other non-anchor vehicles are referred to as agents. To achieve the distributed mode, the vehicles communicate with the available set of neighbors rather than abiding by the pre-configured borders of the network. Hence the network boundaries are not fixed but determined under considerations of communication coverage, signal-to-noise ratio (SNR), and propagation delay. Based on this strategy, the anchor is selected on a consensus basis, where each vehicle possesses the knowledge of the noise level of its sensory devices. With this knowledge, each vehicle can determine if it can serve as the anchor to the neighbors. If present, road-side infrastructures with measurement and communication capabilities can also serve as anchors. Such infrastructures can serve as high-quality anchors because of their deterministic position and possible availability of additional computation and communication resources. For the most intended systems, LTE device-to-device (D2D) protocol of LTE Sidelink [21] is deployed for the message delivery. Under this deployment, vehicles broadcast the messages with source information, while an asynchronous collision avoidance scheduling is done if an infrastructure is present.

The ground truth position of vehicle $v_{i}$, where $i=\{1,2,...,N_{v}\}$, is represented in two-dimensional coordinates denoted by ${\tilde{p}}_{v_{i}}=\left({\tilde{p}}_{x_{v_{i}}},{\tilde{p}}_{y_{v_{i}}}\right)$. The measurements of ${\tilde{p}}_{v_{i}}$ are subject to additive measurement noises of self-localization with the same dimensions denoted by $n_{v_{i}}=\left(n_{x_{v_{i}}},n_{y_{v_{i}}}\right)$. Vehicle $v_{i}$ thus obtains an erroneous measurement $p_{v_{i}}={\tilde{p}}_{v_{i}}+n_{v_{i}}$. Upon the measurement, vehicle $v_{i}$ transmits a message pertaining to $p_{v_{i}}$ to its neighbors via mmWave communication. By the transmission of mmWave, the inter-vehicular distance and AoA with respect to each neighboring vehicle is measured [22,23]. In particular, the distance from vehicle $v_{i}$ to vehicle $v_{j}$ is defined as ${\tilde{d}}_{v_{i},v_{j}}\equiv {\tilde{p}}_{v_{j}}-{\tilde{p}}_{v_{i}}$. In a similar manner to observing $p_{v}$ instead of ${\tilde{p}}_{v}$, additive noise $n_{v_{i},v_{j}}=\left(n_{x_{v_{i},v_{j}}},n_{y_{v_{i},v_{j}}}\right)$ occur. The resulting erroneous measurement is therefore $d_{v_{i},v_{j}}={\tilde{d}}_{v_{i},v_{j}}+n_{v_{i},v_{j}}$.

**Figure 1:**
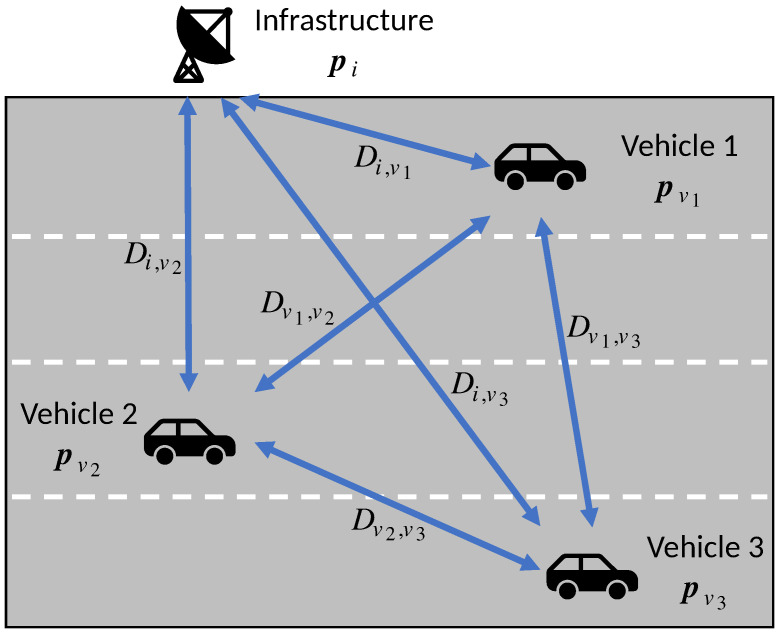
A possible scenario of the vehicle-to-everything (V2X) network.

Figure 1 illustrates an example configuration of the system model. Three vehicles $v_{1}$, $v_{2}$ and $v_{3}$ can respectively observe $p_{v_{1}}$, $p_{v_{2}}$ and $p_{v_{3}}$ by self-localization. The vehicles send their location estimates to each other through mmWave communication. The measurement of the distance and angle of arrival (AoA) to each other is also made. For example, in communication between $v_{1}$ and $v_{2}$, pairs of distance and AoA $d_{v_{1},v_{2}}$ and $d_{v_{2},v_{1}}$ are measured. The figure collectively denotes such pair of distance and AoA measurements as $D_{v_{1},v_{2}}$. The figure also introduces a road-side infrastructure denoted as *i*, with its position denoted by ${\tilde{p}}_{i}$. Due to the infrastructure’s immobility, it is free of positioning error and thus serves as the anchor.

## 3. Proposed Strategy

The strategy for vehicles to cooperatively improve the localization accuracy proceeds in two separate steps: consensus-based updates and compensation. Each step is respectively discussed in detail in the following subsections.

### 3.1. Consensus-Based Update

The first step of the strategy is consensus-based updates. The location estimates of the vehicles are exchanged in a consensus-based and iterative manner. Prior to the initialization of the update, an individual vehicle in the network performs the self-localization. The resulting location estimate is exchanged with each other after the initial measurement is made. These exchanges are not necessarily made in a synchronous manner but only for pairs with pairwise connection available in the network. With the exchanged information at hand, the measurements of distance and AoA with respect to each neighboring vehicle are available. With the collected measurements, new estimates can be calculated by combining the neighboring vehicles’ location estimates and the pairs of distance and AoA with respect to the corresponding partner. In this manner, one new location estimate can be obtained for each neighboring vehicle. If each of $N_{v}$ vehicles can reach to all other neighbors, each vehicle in the network can obtain $N_{v}-1$ new estimates. With the new estimates obtained, the vehicles updates their location estimates to a weighted average of the new estimates and the previous estimates. After the update, exchanges and updates are repeated. With time instances t={0,1,2...} denoted as superscripts, the update of the location estimate of vehicle $v_{i}$ from $p_{v_{i}}^{\left(t\right)}$ to $p_{v_{i}}^{\left(t+1\right)}$ can be described as

(1) $$p_{v_{i}}^{\left(t+1\right)}=\frac{1}{N_{v}}\left\{p_{v_{i}}^{\left(t\right)}+\sum_{v\in N(v_{i})}\left(p_{v}^{\left(t\right)}+d_{v,v_{i}}\right)\right\},$$

where $N(v_{i})$ indicates the set of neighbors of the vehicle $v_{i}$ excluding itself. Note that the coefficient $1/N_{v}$ is valid only when the network is strongly connected or complete, that is, all vehicles are able to communicate with all other neighboring vehicles. If the network is sparse and weighted average is applied, the coefficient can be replaced by an appropriate pair of two coefficients. However, the following analysis can be extended in a straightforward way and the corresponding discussion proceeds analogously. Thus, we stick to the complete network case for simple representation.

For each update, new location estimate $p_{v_{i}}^{\left(t\right)}$ is acquired. With some simple algebra, the analytical expression of the localization error of vehicle $v_{i}$ at an arbitrary time *t* is found in a linear combination of four different types of the measurement noises. Through such decomposition, the localization error at time *t* can be expressed as weighted sums of four different types of noises, as 

(2) $$p_{v_{i}}^{\left(t\right)}-{\tilde{p}}_{v_{i}}=w_{1}n_{v_{i}}+\sum_{v\in N(v_{i})}w_{2}n_{v}+\sum_{v\in N(v_{i})}w_{3}n_{v,v_{i}}+\sum_{v^{\prime \prime }\in N\left(v^{\prime }\right),v^{\prime }\in N\left(v_{i}\right)}w_{4}n_{v^{\prime \prime },v^{\prime }},$$

where $w_{1}$, $w_{2}$, $w_{3}$, and $w_{4}$ indicate the weights on the different types of the measurement noises with respect to the vehicle. The first $w_{1}$ is the weight on the self-localization noise itself. The second $w_{2}$, on the other hand, is the weight on the sums of the self-localization noises of the neighboring vehicles. The last two $w_{3}$ and $w_{4}$ denote the weights of the pair-wise distance and AoA measurement noises, respectively. To be precise, $w_{3}$ is the weight on the sum of noises that occur upon pair-wise measurements from the neighborhood. $w_{4}$ is also the weight on the sum of pair-wise measurement noises, but from the neighborhood of a neighbor to the neighbor. Since the initial measurement noises are exchanged throughout the consensus-based updates, the change in $p_{v_{i}}^{\left(t\right)}$ upon the iterative updates is described by the change of the weights $w_{1}$, $w_{2}$, $w_{3}$, and $w_{4}$ over time. Assume that the self-localization noises that correspond to $w_{1}$ and $w_{2}$ are of zero mean and variance $\sigma_{self}^{2}$, while the means and variances of the pair-wise distance and AoA measurement noises that correspond to $w_{3}$ and $w_{4}$ are also set to zero and $\sigma_{pair}^{2}$, respectively. Although specific values of $\sigma_{self}^{2}$ and $\sigma_{pair}^{2}$ differ by individual vehicles, the expectation of the squared localization error can be collectively expressed as a combination of $\sigma_{self}^{2}$ and $\sigma_{pair}^{2}$ as

(3) $$\begin{array}{cc} E[∥p_{v_{i}}-{\tilde{p}}_{v_{i}}{∥}^{2}] & =\sigma_{self}^{2}\left(w_{1}^{2}+w_{2}^{2}\cdot {\left(N_{v}-1\right)}^{2}+2w_{1}w_{2}\cdot \left(N_{v}-1\right)\right)\\ \; & +\sigma_{pair}^{2}\left(w_{3}^{2}\cdot {\left(N_{v}-1\right)}^{2}+w_{4}^{2}\cdot {\left(N_{v}-1\right)}^{4}+2w_{3}w_{4}\cdot {\left(N_{v}-1\right)}^{3}\right). \end{array}$$

Since the sensory noise levels $\sigma_{self}^{2}$ and $\sigma_{pair}^{2}$ are determined by the qualities of the devices and the environment, the reduction of the localization error by the proposed scheme primarily relies upon the reduction of weights $w_{1}$, $w_{2}$, $w_{3}$, and $w_{4}$. To examine the changes of the weights, update rule of their values upon an iteration can be described as a linear system of equations as

(4) $$w^{\left(t+1\right)}=Aw^{\left(t\right)}+b,$$

where $w={\left[w_{1},w_{2},w_{3},w_{4}\right]}^{T}$ and $b={\left[0,0,N_{v}^{-1},0\right]}^{T}$. A is partitioned as

(5) $$A=\left[\begin{array}{cc} \bar{A} & 0_{2,2}\\ 0_{2,2} & \bar{A} \end{array}\right],$$

where $0_{m,n}$ indicates a *m*-by-*n* zero matrix. In a fully connected network, $\bar{A}$ is given by

(6) $$\bar{A}=\left[\begin{array}{cc} \frac{1}{N_{v}} & \frac{N_{v}-1}{N_{v}}\\ \frac{1}{N_{v}} & \frac{N_{v}-1}{N_{v}} \end{array}\right].$$

The resulting linear system described by (4)–(6) suggests that the weight update rules of pair $\left\{w_{1},w_{2}\right\}$ and pair $\left\{w_{3},w_{4}\right\}$ are independent but similar to each other, with a slight difference of adding $N_{v}^{-1}$ to $w_{3}$. To observe the changes of the weights upon multiple repetitions of the updates, the *n*th power of $\bar{A}$ can be found by the diagonalization of $\bar{A}$. Two eigenvalues $\lambda_{1}$ and $\lambda_{2}$ of $\bar{A}$ are found as 1 and 0, while the corresponding eigenvectors $v_{1}$ and $v_{2}$ are found as ${\left[1,1\right]}^{T}$ and ${\left[1,-{\left(N_{v}-1\right)}^{-1}\right]}^{T}$, respectively. Thus, $\bar{A}$ can be diagonalized as $PDP^{-1}$ where $P=[v_{1}\;v_{2}]$ and the diagonal matrix D is given by

(7) $$D=P^{-1}\bar{A}P=\left[\begin{array}{cc} 1 & 0\\ 0 & 0 \end{array}\right].$$

**Figure 2:**
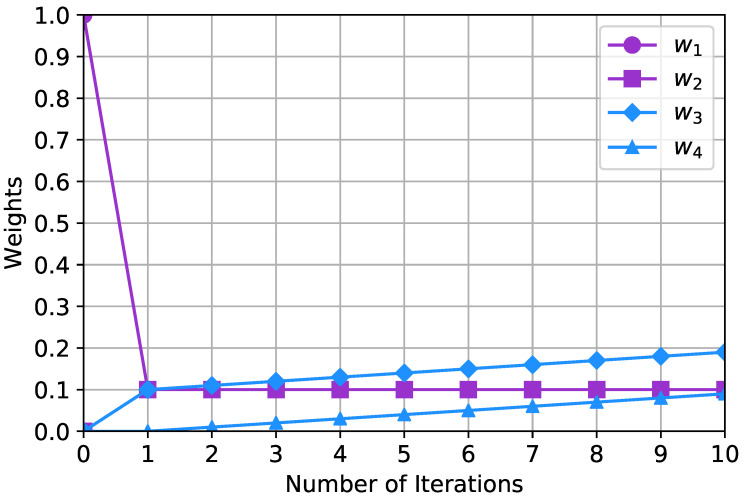
Weight changes upon consensus-based updates.

Noting that ${\bar{A}}^{n}=PD^{n}P^{-1}$, the *n*th power of $\bar{A}$ equals $\bar{A}$ itself. The addition of b in Figure 2 upon the consensus-based update is therefore the only cause of the change of the weights. This indicates that if all vehicles are within each other’s communication range, a single attempt of the update suffices. In other words, the purpose of repeating the updates is in propagating the location estimates throughout the network, not in increasing the localization accuracy.

To observe particular values of weights $w_{1}$, $w_{2}$, $w_{3}$, and $w_{4}$ upon the iteration, further algebraic manipulations obtain closed-form expressions of the weights at time *t*, as

(8) $$\begin{array}{cc} w_{1}^{\left(t\right)}= & w_{2}^{\left(t\right)}=\frac{1}{N_{v}}, \end{array}$$

(9) $$\begin{array}{cc} w_{3}^{\left(t\right)}= & \frac{1}{N_{v}}+\frac{t-1}{N_{v}^{2}}, \end{array}$$

(10) $$\begin{array}{cc} w_{4}^{\left(t\right)}= & \frac{t-1}{N_{v}^{2}}. \end{array}$$

The above expressions show that the update rules on self-localization noises $w_{1}$ and $w_{2}$ immediately converge to $N_{v}^{-1}$. On the other hand, the weights on the pair-wise measurement noises, $w_{3}$ and $w_{4}$ increase linearly. Such a contrast between the pairs of $\left\{w_{1},w_{2}\right\}$ and $\left\{w_{3},w_{4}\right\}$ indicates that the localization accuracy during the consensus-based updates is dependent on the qualities of the pair-wise measurements. In particular, the update of weight $w_{3}$ involves a constant addend $N_{v}^{-1}$ from (4), which then is again added to $w_{4}$ during the subsequent update. As a result, $w_{3}$ and $w_{4}$ are expected to increase by a constant amount of $N_{v}^{-2}$.

Figure 2 illustrates the characteristics of the changes in the weights upon 10 iterations of the updates, where $N_{v}=10$ vehicles are available in a network. At the initial measurement of a vehicle’s own location at step 0, only the noise corresponding to $w_{1}$ exists since neither any exchange of the location estimates nor pair-wise measurement is made. Thus, with no consensus-based update, the value of $w_{1}$ equals unity, while those of $w_{2}$, $w_{3}$, and $w_{4}$ equal zero. Afterwards, as the consensus-based updates are executed, $w_{1}$ is diminished and $w_{2}$ is raised because the initially occurred self-localization error is exchanged and averaged with the neighboring vehicles. On the other hand, $w_{3}$ and $w_{4}$ begin to increase after the exchanges are made. Specifically, the figure shows that $w_{1}$ and $w_{2}$ converge to 0.1 immediately after the first update. This coherently corresponds to the analysis that $w_{1}$ and $w_{2}$ converge to $N_{v}^{-1}$. On the other hand, $w_{3}$ and $w_{4}$ steadily increase by 0.01 as the iteration continues. This trend also appropriately corresponds to the analysis that $w_{3}$ and $w_{4}$ increase by $N_{v}^{-2}$.

In general cases where the self-localization and pair-wise measurement noises occur in random directions, the increase of $w_{3}$ and $w_{4}$ upon the repeated updates lead to the increase in the total amount of localization errors. However, the consensus-based updates are not designed for the immediate reduction of the localization error; the effective reduction of the localization error is achieved by the compensation step described in the following subsection. Instead, the primary aim of the consensus-based updates is to average out the estimate errors of the neighboring vehicles. A proper stopping point for the consensus-based updates is therefore needed since the continued addition of the noises pertaining to $w_{3}$ and $w_{4}$ is rather aimless. The condition to stop the iteration can be found by the collective trend in the change of the location estimates upon a sufficient number of the iteration. The convergence of $\left\{w_{1},w_{2}\right\}$ and the linear increase of $\left\{w_{3},w_{4}\right\}$ cause the location estimates to be updated by similar amounts and directions for all vehicles at each iteration. The repeated addition of similar magnitudes can thus serve as an indicator to halt the iteration. Such a halt condition regarding the amount of the changes of the location estimates can be described in terms of threshold $C_{h}$ as

(11) $$∥p_{v}^{\left(t\right)}-p_{v}^{\left(t-1\right)}∥-∥p_{v}^{\left(t-1\right)}-p_{v}^{\left(t-2\right)}∥\leq C_{h},$$

where a specific value of $C_{h}$ is determined according to conditions of the network. Upon the achievement of (11), the compensation takes place to reduce the localization error effectively.

### 3.2. Compensation

As discussed in the preceding subsection, the noises pertaining to the pairwise measurements steadily increase as the updates are repeated. While this causes the overall magnitude of the localization error to increase, similarities between the remaining localization errors are made. The compensation is designed to counteract the accumulated error by taking advantage of such similarities and the anchor’s superior localization performance.

If the measurement noise has zero mean, the magnitude of the average of the noises is less significant compared to many of the vehicles’ innate noises since noises in the different directions cancel out upon the addition. Since the agents have relatively low measurement accuracy, the agents’ localization estimates become reliable after the consensus-based updates. On the contrary, the anchor initially possesses more accurate location estimate than the agents. During the updates, the anchor receives relatively inaccurate measurements from its neighbors, thus resulting with the aggravated estimate after the updates. These characteristics can be manipulated by undoing the updates of all vehicles with respect to the anchor. In other words, the vehicles’ location estimates can be compensated by $p_{v_{*}}^{\left(0\right)}-p_{v_{*}}^{\left(t_{h}\right)}$, where $v_{*}$ and $t_{h}$ denote the anchor and the time of the iteration halt, respectively. Apparent is the restoration of the anchor’s localization to the initial and superior state. Since all vehicles’ estimates contain similar magnitude and orientation of error, the compensation brings not only the anchor’s but also all agents’ location estimates closer to the ground truth locations. The compensation step can thus simultaneously bring down the localization errors within the network.

Continuing from the analysis of the previous subsection, the compensated estimate of vehicle $v_{i}$, denoted by $p_{v_{i}}^{\left(comp\right)}$, is expressed as

(12) $$p_{v_{i}}^{\left(comp\right)}={\tilde{p}}_{v_{i}}+\frac{1}{N_{v}}\sum_{v\in N(v_{i})}n_{v,v_{i}}-\frac{1}{N_{v}}\sum_{v\in N(v_{*})}n_{v,v_{*}}+n_{v_{*}}.$$

With the above expression of the estimate, the CRLB of the estimator can be found as

(13) $$E[∥p_{v_{i}}^{\left(comp\right)}-{\tilde{p}}_{v_{i}}{∥}^{2}]\geq \frac{1}{I(\sigma_{v_{*}})}=2\sigma_{v_{*}}^{2}.$$

The corresponding Fischer information $I(\sigma_{v_{*}})$ is given by

(14) $$I\left(\sigma_{v_{*}}\right)=-E\left[\frac{\partial^{2}}{\partial {\tilde{p}}_{v_{i}}^{2}}\ln L\left({\tilde{p}}_{v_{i}}|p_{v_{i}}^{\left(comp\right)},\sigma_{v_{*}}\right)\right]=\left[\frac{1}{\sigma_{v_{*}}^{2}},\frac{1}{\sigma_{v_{*}}^{2}}\right],$$

where $L({\tilde{p}}_{v_{i}}|p_{v_{i}}^{\left(comp\right)},\sigma_{v_{*}})$ denotes the likelihood of the estimator given by

(15) $$L\left({\tilde{p}}_{v_{i}}|p_{v_{i}}^{\left(comp\right)},\sigma_{v_{*}}\right)=\frac{1}{2\pi \sigma_{v_{*}}^{2}}\exp\left(-\frac{1}{2\sigma_{v_{*}}^{2}}{\left(p_{v_{i},x}^{\left(comp\right)}-{\tilde{p}}_{v_{i},x}\right)}^{2}-\frac{1}{2\sigma_{v_{*}}^{2}}{\left(p_{v_{i},y}^{\left(comp\right)}-{\tilde{p}}_{v_{i},y}\right)}^{2}\right).$$

The resulting CRLB in (13) indicates that the accuracy of the compensated estimate largely depends upon the accuracy of the anchor.

### 3.3. Practical Implementation

In the compensation phase, the anchor is responsible for the broadcast of the compensation information to the network. However, for a single broadcast to suffice, the connections among all pairs of vehicles are required. Some vehicles nevertheless may not be able to successfully receive information from the anchor because of the lack of direct connections. Furthermore, temporary connection malfunction between the anchor and the agents causes the failure in the compensation. In the current description of the proposed strategy, the vehicles postpone the next update until other vehicles receive the compensation successfully. Forcing a synchronous operation in such a way diminishes the purpose of the distributed algorithm.

To overcome these issues, a slightly modified and practical method of conveying the compensation information is used. In the practical implementation, instead of waiting for the iteration halt and calculating the overall change in the location estimate, the anchor may execute a bit of the compensation upon each iteration. In other words, the anchor compares its location estimates before and after each update and then broadcasts the difference to the neighboring vehicles. The vehicles that successfully receive the broadcast then possess a portion of the compensation, which is repeatedly exchanged with other vehicles upon the next update. Through this modification, each vehicle asynchronously perform its updates regardless of receiving the broadcast from the anchor. Such an asynchronous mode can enhance the robustness to the connection failures or short communication ranges. Furthermore, the practical implementation does not degrade the accuracy of the protocol. In fact, when some single-hop distances are greater than the communication range, the practical can implementation enhance the accuracy by propagating the compensation throughout the network upon the repeated updates. The modification thus achieves the asynchronous operation without the performance loss or the restriction to applicability. The practical implementation of the proposed strategy with respect to vehicle $v_{i}$, with the complexity of $O(|N\left(v_{i}\right)|)$, is summarized in Algorithm 1.
**Algorithm 1** Strategy of improvement of localization of $v_{i}$  1: At time t=0 initialization  2: Measure $p_{v_{i}}^{\left(t\right)}$  3: **while** (11) does not hold **do**  4:      t⇐t+1  5:      Receive $p_{v}^{\left(t-1\right)}$ and measure $d_{v_{i},v}$ from $v\in N(v_{i})$  6:      Calculate $p_{v_{i}}$ by (1)  7:      **if** broadcast from anchor received **then**  8:            $p_{v_{i}}^{\left(t\right)}⇐p_{v_{i}}+p_{v_{*}}^{\left(t-1\right)}-p_{v_{*}}^{\left(t\right)}$  9:      **else**  10:          $p_{v_{i}}⇐p_{v_{i}}^{\left(t\right)}$  11:    **end if**  12: **end while**

It is noted that protocol-specific design details of the proposed strategy depend upon the choice of specific communication types. While this work aims for generic LTE V2X systems, tuning of the strategy and its parameters, such as latency, distance of reliable connection between two vehicles, and frequency of the messages, are properly modified according to the specific use cases.

## 4. Experiment

To evaluate the performance of the proposed strategy in details, a 1300 m × 750 m vehicular network consisting of 20 vehicles is simulated. The vehicles perform self-localization under additive Gaussian noise of σ=25 m. Note that the noise level of the self-localization of the agent vehicles have no direct effect on the performance of the proposed strategy. Thus, a rather large setup of σ=25 m is for the purpose of lucid observation of the changes of the location estimates. On the other hand, one of the 20 vehicles has the most accurate means of self-localization and thus serves as the anchor. The self-localization performance of the anchor is decisive to the effectiveness of the proposed strategy. The location of the anchor is assumed to be deterministic in this case for the purpose of clear illustration of the strategy. Such a deterministic location of the anchor can be achieved when a road-side unit (RSU) [24] with measurement and communication capabilities is available. General cases where the measurement of the anchor is not very accurate are discussed in the subsequent section. While using mmWave-based technologies, additive zero-mean Gaussian noises with σ=1 m and $\sigma =3^{\circ }$ occur during measurements of pair-wise distance and AoA, respectively. For the tested network size, the propagation delay can extend up to 5.00 ms if the communication distance is not limited. To achieve at least three message exchanges within 10 ms, the maximum communication distance is set to 900 m in the simulations unless specified otherwise. General choices of all simulation parameters follow the configuration of the urban scenario specified in 3GPP TR 37.885, while the performances under different circumstances are discussed briefly.

**Figure 3:**
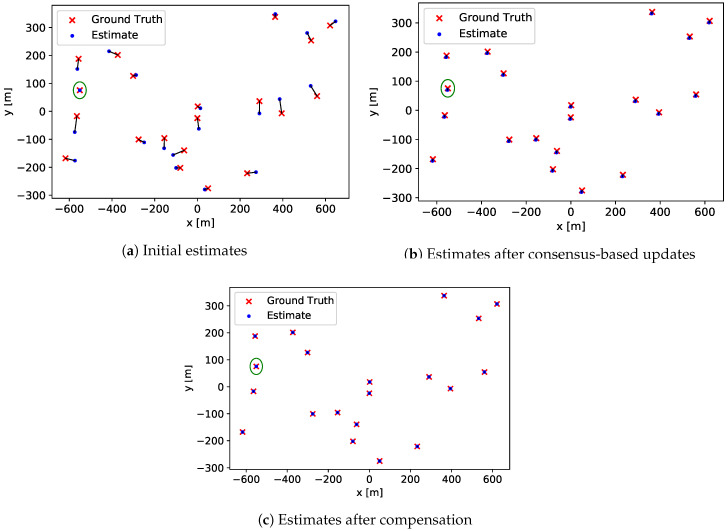
Changes of estimates during the execution of the proposed strategy.

Figure 3 illustrates changes of the location estimates during the overall execution of the proposed strategy. Unlike the practical implementation discussed in Section 3.3, the compensation step is illustrated separately in order to clarify its role. The effect of adopting the practical implementation is subsequently discussed. The comparison is made between the ground truth locations and the estimates at three different stages—the initial estimation, the halt of the consensus-based updates, and the compensation. Each pair of the real and the estimated locations is connected by a line for the unambiguity. The estimate and the ground truth location of the anchor are indicated by a surrounding circle. Figure 3a shows the initial stage of the strategy, where no update has been applied on the estimates. Self-localization errors misplace the estimates randomly with respect to each corresponding ground truth locations. Since the self-localization of the anchor is noiseless in this particular case, the estimate made by the anchor is correctly placed onto the ground truth location. On the other hand, the immediate purpose of the iteration of the update is the delivery of the messages to as many neighbors as possible, rather than the convergence to an accurate value. To meet this purpose, the exact value of the threshold is chosen so that enough number of multi-hop can occur for the farthest pair of neighbors in the network can exchange messages. For the proposed test case, the threshold of $C_{h}=0.5$ m suffices for the longest possible inter-vehicular length of 1500 m. Under such a halt condition, four updates are iterated before the halt. The result of the consensus-based updates are shown in Figure 3b. The updated estimates are all similarly misplaced slightly downwards and to the left with respect to each corresponding real location. This shows that the updated estimates of all vehicles carry similar magnitudes and orientations of the error. Figure 3c depicts the location estimates after the compensation is applied. As indicated by the estimates placed onto the ground truths, much accurate estimates are achieved after the compensation.

With the adoption of the practical implementation, each iteration of the update includes a portion of the compensation. When the update is halted by the convergence of the estimates, all portions of the compensation is conveyed to all vehicles in the network. If the practical implementation of the proposed strategy is applied under the identical system settings, the estimates therefore converge to the ones shown in Figure 3c instead of the ones in Figure 3b.

**Figure 4:**
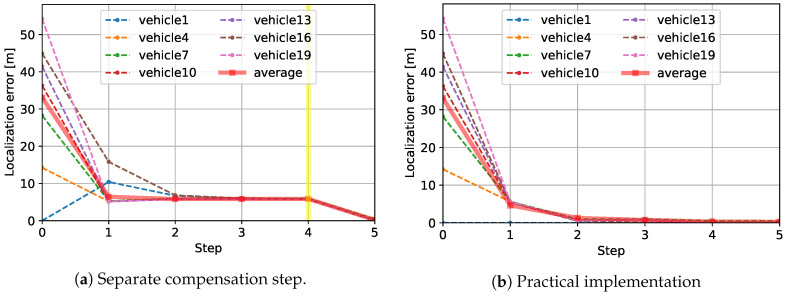
Measurement errors at each step.

For the same test case, the reduction of the localization error at each step is shown in Figure 4 to compare both implementations of the compensation. Figure 4a shows the strategy where the consensus-based updates and the compensation are separately applied, while Figure 4b shows the strategy under the practical implementation. For clear representation, only a subset of 20 vehicles’ localization errors are presented; the vehicles are sorted by the amount of initial localization error and then every third vehicles are selected. Thus, the localization errors of 7 out of 20 vehicles are represented by the dashed lines. In addition, the average localization error of all 20 vehicles is shown together by the solid red lines. At the initial step, the amounts of the localization errors highly differ from each other. For instance, the anchor vehicle represented by the blue dashed line has no initial localization error while that of another vehicle is higher than 50 m. Such randomness rapidly diminishes in the following steps. First, in Figure 4a, as the consensus-based updates are executed from step 1 to step 4, the magnitudes of the vehicles’ localization errors are gradually homogenized. In particular, from step 2 to 4, little changes in the localization errors are observed as measurement noises are averaged out on the consensus basis. A yellow vertical line at step 4 indicates that the halt condition (11) for the consensus-based updates is met at the fourth update. At step 5, the compensation reduces the remaining localization errors effectively, leaving the estimates much more accurate than before. On the contrary, Figure 4b makes no distinction between the consensus-based updates and the compensation. While most features regarding the reduction of errors are retained, it is noted that the localization errors achieved in Figure 4a are also achieved in Figure 4b, indicating that the practical implementation of the compensation step does not degrade the localization performance. The performances in more diverse scenarios are discussed in the following section.

## 5. Evaluation

The experiment on specific setup of the previous section poses questions regarding how changes in the system setup affects the performance of the localization strategy. For comprehensive comparison of the effect of the proposed strategy under diverse circumstances, the improvement factor (IF) is introduced as

(16) $$IF=\frac{\mathrm{initial}\;\mathrm{average}\;\mathrm{localization}\;\mathrm{error}-\mathrm{final}\;\mathrm{average}\;\mathrm{localization}\;\mathrm{error}}{\mathrm{initial}\;\mathrm{average}\;\mathrm{localization}\;\mathrm{error}}.$$

**Figure 5:**
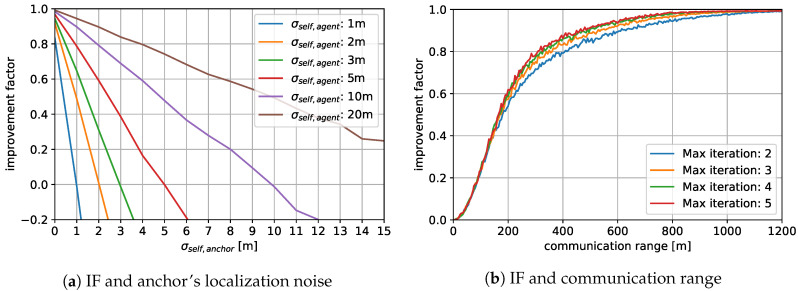
Changes of improvement factor (IF) of the proposed strategy under varying circumstances.

Under this definition, IF=1 if the proposed strategy completely removes the localization error. Also, IF=0 if it does not change the localization at all, and IF<0 if it degrades the localization and the proposed strategy should rather not be used. Figure 5 shows the changes of IF under multiple varying system parameters such as measurement noises and connection statuses between the vehicles. It is noted that the noise level of the pairwise AoA measurement has little to no direct impact on the performance and thus is fixed to $3^{\circ }$. Such an observation is made since, when the directional information are averaged during the consensus-based updates, the magnitude of the resulting AoA does not hold any significant information and does not affect the preciseness of the localization.

Figure 5a shows how IF changes with respect to the noise level of self-localization, denoted by $\sigma_{\mathrm{self},\;\mathrm{anchor}}$. The change is observed under different settings of the agents’ self-localization noise levels, similarly denoted as $\sigma_{\mathrm{self},\;\mathrm{agent}}$. The increase of the self-localization error has a negative linear effect on IF since the trend of IF according to the noise level can be approximated to a straight line. For each line, the point where IF=0 is reached when $\sigma_{\mathrm{self},\;\mathrm{anchor}}=\sigma_{\mathrm{self},\;\mathrm{agent}}$. For example, when $\sigma_{d}$ of the agents equal 5 m, the corresponding red line crosses the point where IF=0 when $\sigma_{d}$ of the anchor equals 5 m. This suggests that the presence of an anchor that is superior to the agents in terms of the self-localization guarantees the improvement in the localization performance of all vehicles in the network. In other words, as long as the vehicle of the best localization accuracy can be properly determined, the proposed strategy enhances the localization accuracy of all vehicles. On the other hand, when $\sigma_{d}$ of the agents are sufficiently low, IF does not reach 1 regardless of the precision of the self-localization of the anchor. This does not necessarily indicate that the estimates are less accurate when agent vehicles are capable of sufficiently precise localization; with lesser amount of noise is initially present, the proportion of the remaining noise is larger in such cases, thus preventing the IF from reaching 1.

Figure 5b shows the change of IF upon varying communication ranges. With insufficient communication range, not all neighboring vehicles may be able to directly communicate with each other. This prevents thorough delivery of all local information within the network. Since the consensus protocol takes advantage of abundant local data, the lack of the full information leads to degradation of the accuracy. However, the receipt of estimates from long distances may also deteriorate the accuracy somehow. The measurement error becomes larger when two vehicles are farther from each other since the same AoA measurement error can cause larger positional error at greater distances. Furthermore, signals tend to degrade over longer ranges and thus aggravates this issues. While the expansion in the communication range results in the availability of more local information, additional information are therefore relatively inaccurate and may be undesirable. These suggest a possible trade-off between the quantity and the quality of the measurement. Nonetheless, a longer communication range increases the IF, suggesting that the benefit of the availability of more estimates overcomes the possible inflow of more erroneous data. In addition, the loss of the accuracy caused by short communication range is overcome by the increased number of the iteration of the updates; Local information that is too far from a vehicle to be reached with a single hop is ultimately delivered via multi-hop transmission. Under the same communication range, higher IF is achieved by allowing more iteration.

**Figure 6:**
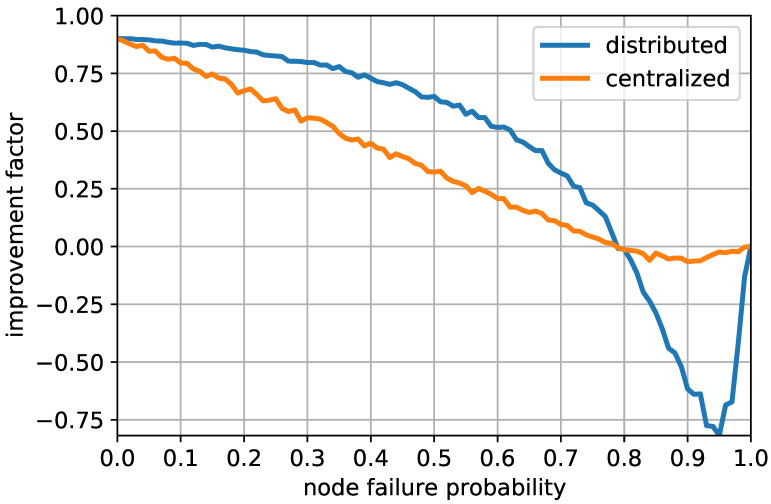
Comparison with centralized approach.

While Figure 5 shows the changes in the efficiency of the proposed strategy under various circumstances, Figure 6 shows whether the distributed implementation indeed grants more accuracy than simply employing a centralized management. For detailed comparison, situations where vehicles may be temporarily unavailable due to communication error are simulated. For the distributed protocol, when the anchor fails to communicate with others, an alternative vehicle temporarily acts as an anchor. The substitute for the anchor is chosen by $\sigma_{self}^{2}$, the noise level of the sensory devices that pertain to the self-localization. To represent accuracy differences among the vehicles, 20 different choices of $\sigma_{self}$ are set to 1 m, 2 m..., 20 m. In such configurations, when the vehicle with $\sigma_{self}^{2}=1$ m${}^{2}$ fails to communicate, another available vehicle with the lowest $\sigma_{self}^{2}$ temporarily acts as an anchor. On the other hand, for the centralized implementation, only the central manager can serve as an anchor. When the central manager fails to communicate, all vehicles in the network are thus unable to perform the update, leaving with the unchanged initial estimates. The IF of both the distributed and the centralized approaches are presented according to the node failure probability ranging from 0 to 1. In both cases, zero node failure probability indicates that all vehicles stay available for the respective protocols. Since both implementations proceed in the same steps when no node failure occurs, both curves start at the same IF. As the node failure probability increases, the distributed protocol has a high IF over the centralized protocol. This suggests that as communication error occur more often, the distributed implementation successfully adapts to temporary setbacks while the centralized implementation cannot. However, When the node failure probability exceeds 0.8, both distributed and centralized implementations result in IF below 0, suggesting that the vehicles’ initial self-localization results are more accurate than the results that utilize poorly exchanged data. In addition, the distributed implementation shows far worse performance than the centralized implementation when the node failure probability is between 0.8 and 1. This observation is made since, when most vehicles become unavailable during the exchange, the distributed protocol may send out the compensation based on the vehicle of very poor accuracy, thus degrading the quality of all estimates in the network. Finally, if the node failure probability equals 1, no exchange is carried out and both IFs equal 0 at this point. The comprehensive observation thus suggests that the distributed implementation outperforms the centralized one for reasonable range of the node failure probability and that utilization of the proposed strategy may be undesirable when the communication is severely impeded.

**Figure 7:**
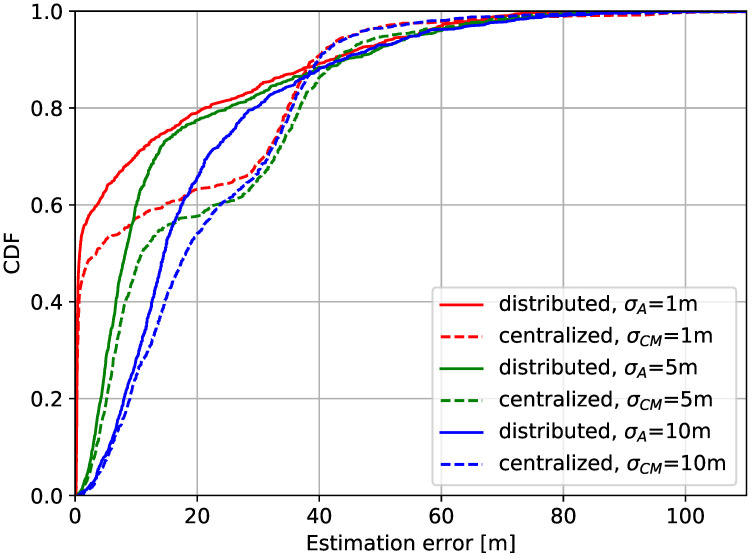
Cumulative distribution function of the localization error of the proposed strategy.

Figure 7 illustrates cumulative distribution functions (CDF) of the localization errors under different qualities of the anchor. The CDFs for multiple set of measurement noise levels are shown for both the distributed and the centralized modes. For the distributed mode, the measurement noise level of the anchor is denoted as $\sigma_{A}^{2}$, where that of the central manager is denoted as $\sigma_{CM}^{2}$. For thorough observation, multiple probabilities of the communication failure were sampled from range of 0 to 0.8. Each pair of $\sigma_{A}^{2}$ and $\sigma_{CM}^{2}$ of equal values are categorized by the same color of the graph. The figure shows that, for each pair of the measurement noise levels, the CDFs of the distributed protocol increase more rapidly than the those of the centralized protocol at lower estimation errors below 40 m. This indicates that the distributed mode exhibits more localization results with lower estimation errors. However, for estimation errors above 40 m, the CDFs of the distributed mode converge more slowly to 1 compared to those of the centralized mode. This indicates that low quality measurements of the anchor can be propagated throughout the network in the distributed mode, degrading the overall quality of the localization. While the distributive localization can provide high-accuracy estimation in most cases, the message exchanges are therefore inhibited when measurements of such low qualities are made in order to contain erroneous measurement.

**Figure 8:**
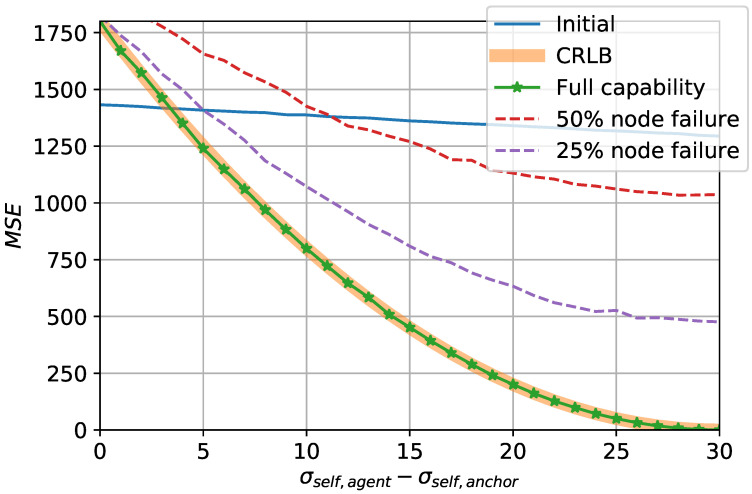
Comparison of Cramér-Rao lower bound (CRLB) and the proposed strategy.

To observe whether the proposed strategy achieves the optimal performance, Figure 8 compares the CRLB obtained in (13) and the mean squared error (MSE) of the resulting location estimates under different circumstances. The horizontal axis of the figure indicates the difference between the noise levels of the self-localization of the anchor and the agents, respectively denoted by $\sigma_{\mathrm{self},\mathrm{anchor}}$ and $\sigma_{\mathrm{self},\mathrm{agent}}$. With $\sigma_{\mathrm{self},\mathrm{agent}}$ fixed to 30 m, $\sigma_{\mathrm{self},\mathrm{anchor}}$ decreases from 30 m to 0 m along the axis. The figure shows that the MSE of the proposed strategy under no hindrance, labeled as ’full capability,’ coincides with the CRLB. This indicates that the proposed strategy successfully achieves the optimal performance of the estimator when no particular impediment exists. However, as examined from the previous observations, unfavorable settings may result with updates that are less accurate than the initial estimates. Specifically, when the difference $|\sigma_{\mathrm{self},\mathrm{anchor}}-\sigma_{\mathrm{self},\mathrm{agent}}|$ is less than about 3 m, the minimum achievable MSE of the estimator given by the CRLB is higher than the MSE of the initial estimates. This again validates in a new perspective that in order to benefit from the proposed strategy, anchor’s superiority of preciseness over the agents is required.

The MSE of the proposed strategy under communication setbacks are also compared with the CRLB. In particular, results under 25% and 50% node failure probabilities are shown, respectively. As can be intuitively deduced, the proposed strategy is strays farther from the optimal performance as communication failures occur more often. The initial MSE can also be compared with the MSE of these particular cases to determine whether the utilization of the proposed strategy is desirable.

## 6. Conclusions

A strategy to improve the accuracy of the localization estimate in V2X networks in a consensus-based manner is developed and analyzed. Furthermore, a theoretically intuitive two-step implementation of the proposed strategy is presented and compared in terms of the protocol and the accuracy. The expected accuracy of each step of the strategy is analyzed and the CRLB of the resulting estimator is obtained. While the computations of the strategy are kept very undemanding, the estimator nevertheless achieves its optimum given by the CRLB. The time requirement to fulfill the localization enhancement is thus mainly determined by the propagation delay adjusted by a threshold to the communication distance. Achievement of the optimal estimator is verified by the simulation results under various configurations. Additional insights on the performance changes under various setups are made. With reasonable amount of handicaps, the proposed strategy deviates from the optimum but yet successfully enhances the localization accuracy. The decision making upon the employment of the proposed strategy based on the comparison with the initial MSE is also investigated.
